# Neurodegenerative and Cognitive Consequences of Long COVID

**DOI:** 10.3390/ijms27156897

**Published:** 2026-08-01

**Authors:** Luiza Marek-Józefowicz, Alina Borkowska, Bogusław Nedoszytko, Wiesław Jerzy Cubała, Dorota Purzycka-Bohdan, Michał Bohdan

**Affiliations:** 1 Department of Dermatology, Sexually Transmitted Diseases and Immunodermatology, Dr. Jurasz University Hospital in Bydgoszcz, 9 Marii Sklodowskiej-Curie, 85-094 Bydgoszcz, Poland; lui06@interia.pl; 2 Faculty of Health Sciences, Collegium Medicum, The Mazovian University in Płock, 09-402 Płock, Poland; alinaborkowska361@gmail.com; 3 Fahrenheit Biobank BBMRI.pl, Department of Medical Laboratory Diagnostics, Medical University of Gdansk, Debinki Street 7, 80-211 Gdansk, Poland; boguslaw.nedoszytko@gumed.edu.pl; 4 Department of Dermatology, Venereology and Allergology, Medical University of Gdansk, 80-214 Gdansk, Poland; 5 Department of Psychiatry, Faculty of Medicine, Medical University of Gdansk, Debinki St. 7 Build. 25, 80-952 Gdansk, Poland; cubala@gumed.edu.pl; 6 Clinical Physiology Unit, Medical Simulation Centre, Medical University of Gdańsk, 80-210 Gdansk, Poland; purzycka-bohdan@gumed.edu.pl; 7 First Department of Cardiology, University Clinical Centre, Medical University of Gdansk, 80-210 Gdańsk, Poland

**Keywords:** COVID-19, cognitive impairment, neurodegenerative diseases

## Abstract

The SARS-CoV-2 virus has infected approximately 778 million people worldwide since the pandemic. Patients who have survived coronavirus disease (COVID-19) may experience long-term symptoms related to cognitive deficits, mood changes, and depressive disorders. The mechanisms underlying the long-term effects of COVID-19 on the brain are being actively investigated. SARS-CoV-2 infection triggers various mechanisms, such as hyperstimulation of the immune response, which may lead to changes in the central nervous system. In this article, we review the evidence linking COVID-19 to neurodegenerative disorders and cognitive impairment. Current research indicates that patients with pre-existing cognitive and neuropsychiatric deficits have a poorer prognosis after SARS-CoV-2 infection, and patients who have survived COVID-19 may be at increased risk of developing dementia and mood disorders. We analyse the available evidence regarding SARS-CoV-2 brain infection, induction of inflammation, coagulopathy, and blood–brain barrier (BBB) dysfunction as possible mechanisms underlying the disorders in the acute phase of COVID-19 disease and contributing to the development of neurodegenerative disorders in long COVID. Viral infection can trigger inflammation of the central nervous system (CNS), leading to damage and contributing to the development of cognitive dysfunction.

## 1. Introduction

### 1.1. The Aim

The aim of this manuscript is to present and highlight the impact of SARS-CoV-2 on patients suffering severe symptoms and the risk of developing neuropsychiatric disorders and neurodegenerative diseases. Possible pathomechanisms of changes in the central nervous system are discussed, including the role of the blood–brain barrier, the NLRP3 inflammosome, cytokine storms and their effects on the brain, and potential therapeutic targets in patients with long COVID.

### 1.2. Materials and Methods

This review mainly includes peer-reviewed articles published up to March 2026, coming from databases such as PubMed and Web of Science. A total of 156 items of literature were cited, including original studies, reviews, clinical cohort studies, post-mortem analyses, experimental models on psychoneuroimmunology, cognitive impairment, and neurodegeneration in long COVID.

Human single-stranded RNA coronavirus (CoV) was identified for the first time in 1966 by Tyrell and Bynoe [1]. Coronavirus SARS-CoV-2 entered the human population in Wuhan, China, in late 2019, and is responsible for the development of COVID-19 [2,3]. According to the WHO, it has infected ca. 778 million people worldwide and has led to over 7 million deaths [4]. The SARS-CoV-2 virus can infect people and animals. The spike glycoprotein (S protein) binds to the host cell surface receptor angiotensin-converting enzyme (ACE2), which is most commonly found in alveolar epithelial cells [5]. During the COVID-19 pandemic, 15–20% of hospitalized patients experienced interstitial pneumonia leading to acute respiratory distress syndrome (ARDS). SARS-CoV-2 induces a pathological immunological response that resembles cytokine release syndrome (CRS), resulting in increased expression of pro-inflammatory cytokines such as IL-6 and IFN-γ and the activation of NF-κB and JAK-STAT signalling pathways. Consequently, CRS leads to epithelial and endothelial cell apoptosis, which is an abnormal response of T-lymphocytes leading to lung injury [6,7].

The COVID pandemic has revealed long-term neuropsychiatric sequelae of acute SARS-CoV-2 infection [8]. According to the WHO, in adults, a post-COVID-19 condition occurs in individuals with a history of probable or confirmed SARS-CoV-2 infection, usually 3 months from the onset of COVID-19, with symptoms that last for at least 2 months and cannot be explained by an alternative diagnosis. Common symptoms include fatigue, shortness of breath, and cognitive dysfunction, and generally have an impact on everyday functioning. They may also fluctuate or relapse over time. However, in children, the post-COVID-19 condition, compared with controls, involves fatigue, altered smell/anosmia, and anxiety. Symptoms generally have an impact on everyday functioning, such as changes in eating habits, physical activity, behaviour, academic performance, social functions (interactions with friends, peers, and family) and developmental milestones. Both adult and child symptoms may be new onset following initial recovery from an acute COVID-19 episode or persist from the initial illness. They may also fluctuate or relapse over time [9].

By definition, it is clear that long COVID is a diagnostically challenging process. There are no clear biomarkers for its assessment, making it difficult to assess whether the symptoms a patient presents with are the result of long COVID or another disease entity.

According to existing meta-analyses, the incidence of long COVID ranges from 10% to 43% worldwide and is observed in 54% of people requiring hospitalization and in 34% of those who did not require hospitalization [10,11,12,13].

However, since 2020, there has been a decline in the incidence of long COVID. In the USA, 8.9% of patients (10.3% of women and 7.3% of men) who were infected reported persistent symptoms of long COVID, with the peak age being 30–59 years [14]. It can develop in patients after a severe infection requiring intensive care, as well as in patients after an asymptomatic and mild infection. Any SARS-CoV-2 infection can develop long COVID [15]. Patients with a more severe infection are at higher risk of developing long COVID, which is likely related to the occurrence of a cytokine storm and acute inflammation.

COVID-19-associated neuroprogression is a dynamic disorder of the central nervous system (CNS) in which neuroinflammatory and neurovascular damage are responsible for abnormal frontal–limbic control, clinically manifested by cognitive, depressive, and anxiety disorders [8].

### 1.3. The Impact of Long COVID Varies from Mild Impairment to Severe Disability

As a result of COVID-19 infection, the immune system is modulated [16], resulting in changes in neuroimmune interactions that affect the CNS.

As a result of the activation of the immune system, pro-inflammatory cytokines IL-6 and TNF-α, and vascular endothelial growth factor VEGF are produced, and immunoglobulins are deposited on the vascular endothelium, which leads to its damage and loss of integrity. As a result, the blood–brain barrier (BBB), which protects the CNS from systemic circulation, becomes more porous and permeable, and the brain is exposed to the action of cytokines and reactive oxygen species, leading to the activation of glial cells, exhaustion of T cells, and development of neuroinflammation [17,18,19].

SARS-CoV-2 spike proteins interact with the human angiotensin-converting enzyme 2 (ACE2) receptor, along with accessory molecules that facilitate viral entry, through the spike receptor-binding domain (RBD). Although ACE2 is the primary receptor required for viral replication, its expression patterns do not fully correlate with viral distribution or tissue pathology. Furthermore, SARS-CoV-2 has been shown to infect cells and tissues without detectable ACE2 expression. Viral entry through the ACE2-independent pathways may also confer resistance to some monoclonal antibodies (Abs) directed against spike RBDs, which block ACE2-mediated binding [20]. As a result, the virus enters the host cell and replicates. A decrease in lysozyme activity leads to protein aggregation in neurons and may contribute to the development of neurodegenerative diseases [21].

Post-mortem examination of patients infected with SARS-CoV-2 has revealed the presence of the virus in brain capillaries, endothelial cells, pericytes and neurons [22,23].

However, in the severe course of COVID-19, hypoxia, dysfunction of the lungs and other organs, reduced blood pressure, and metabolic disorders that can negatively affect nerve cells may occur. There are different mechanisms of nervous system damage that can co-occur in patients with different frequencies. The most common mechanism of neuroinflammation is the immune response to a respiratory infection leading to impaired neuronal plasticity. This can occur after a mild course of COVID-10. A rare mechanism is direct infection of the brain by SAR-CoV-2 [24].

## 2. Cytokine Release Syndrome (CRS)—Cytokine Storm in COVID-19

Cytokine release syndrome is caused by the pathological inflammatory response initiated by macrophages, dendritic cells, NK cells, and T cells in response to pathogen-associated molecular patterns [6,25]. Activation of lung capillary endothelial cells may contribute to enhanced immunological response and predispose to cytokine storm [26]. In patients with severe COVID-19, a significant decrease in CD8+ T cells, CD4+, B cells and NK cells [6] and marked elevation of IL-1β, IL-2, IL-4, IL-6, IL-7, IL-8, IL-10, IFN-α/β/γ, MCP-1, CCL-3, CCL2, CCL5, and YKL-40 [27] were found [26,28,29,30]. INF-γ may initiate cytokine storm in patients with SARS-CoV-2 [6,31]. IL-6 and TNF-α are considered major components associated with increasing cytokine storm.

## 3. The Role of the NLRP3 Inflammasome in SARS-CoV-2 Infection

Damage to type II alveolar epithelia expressing ACE2 activates PAMPs (pathogen- associated molecular patterns induced by bacterial, viral, and fungal pathogens) as well as DAMPs (damage-associated molecular patterns from urate crystals and reactive oxygen species) of the NLRP3 cytosolic inflammasome (NOD-like receptor family, pyrin domain-containing 3). Inflammasomes belong to intracellular complexes located in the cytosol; they are part of non-specific immunity and are responsible for detecting molecular patterns indicative of cell damage as well as the presence of pathogens causing infection [32,33]. Their activation is necessary for the stimulation and maturation of cytokines that determine an efficient immune response, leading to virus elimination. The NLRP3 inflammasome plays an important role in host antiviral defence, and its abnormal activation and induction of downstream mediators lead to pathological tissue damage during infection. The developing inflammasome releases the enzyme-caspase1, which participates in cell death or the conversion of inactive pro-inflammatory cytokines into their active forms (IL-1β and IL-18). IL-1β increases the expression of NF-κB, a mitogen-activated protein kinase (MAPK) that stimulates the production of other pro-inflammatory cytokines. The NLRP3 inflammasome is a major inflammatory component involved in the innate immune response when induced by various stimuli [34].

SARS-CoV-2-infected pneumocytes release alveolar macrophages secreting TNF-α and IL-β and initiate a pro-inflammatory cascade [35]. These cytokines induce cell damage and death, activate the NLRP3 inflammasome, and create a hyper-inflammatory microenvironment localized to the tissue injury site; subsequently, pro-inflammatory cytokines are present in the blood due to vascular integrity loss [26,36].

## 4. The Role of the Receptor for Angiotensin-Converting Enzyme ACE2

The strong pro-inflammatory potential of the SARS-CoV-2 spike protein is demonstrated by its ability to bind to ACE2 receptors on microglia, which activates the NLRP3 inflammasome, resulting in the secretion of pro-inflammatory cytokines, intensifying neuroinflammation [37].

Neuroinflammation is inflammation of the central nervous system. The main cells responsible for this condition are microglia and astrocytes. Upon NF-κB activation, microglia increase the transcription of genes to produce more substances that trigger inflammation, such as iNOS, COX-2, TNF-α, IL-6, IL-1, and ROS. Brain inflammation is a major cause of many neurodegenerative diseases, such as Parkinson’s disease (PD) and Alzheimer’s disease (AD) [38].

Neurodegeneration is a complex process underpinning the decline in neuronal functions and synaptic connections in the brain. It is often associated with ageing and usually leads to death. In neurodegeneration, certain proteins in the brain malfunction, causing clusters to form in various parts of the brain. Other problems caused by neurodegeneration, such as inflammation, energy deficiency, and DNA damage, gradually increase in intensity, resulting in cell death. Therefore, it is important to find ways to inhibit neurodegeneration by targeting this inflammation [38].

A large number of ACE2 receptors are found in intestinal epithelial gland cells, the respiratory tract epithelium, lung parenchyma [39,40], and in the immune system and brain [41]. SARS-CoV-2 binding to ACE2 on neurons can disrupt their mitochondrial function and protein folding, contributing to brain degeneration even decades after the index infection [42,43].

## 5. Vascular Endothelial Dysfunction and Blood–Brain Barrier (BBB) Damage

Long-term neuropsychiatric consequences result from changes associated with cytokine storm, which can cause blood–brain barrier (BBB) damage, vascular dysfunction, and punctate infarcts in the brain [24,44]. SARS-CoV-2 can infect the patient’s brain and cause CNS lesions and cognitive impairment. Cranial nerves such as the olfactory, trigeminal, optic, and vagus nerves and the blood–brain barrier (BBB) are important structures for the interaction between SARS-CoV-2 and the brain. The physical barrier between nerve cells and circulating immune cells is the blood–brain barrier (BBB), consisting of brain endothelial cells, vascular basement membrane, astrocytes, microglia, and neurons, known as neurovascular units (NVUs) [39,45,46]. Dysfunction of the blood–brain barrier as a result of infection affects neuronal activity in the CNS and may be related to cognitive impairment and COVID-19 severity.

Once across the BBB, the virus invades brain cells by binding the S1 subunit of the S protein to ACE2 receptors, resulting in a conformational change in the S2 subunit to achieve membrane fusion with the host cell [47]. As a result of SARS-CoV-2 infection, the concentration of pro-inflammatory cytokines and coagulation factors such as thrombin, fibrinogen and plasmin increases, which induces hypoxia [39]. Elevated levels of pro-inflammatory factors interfere with the activity of TJ proteins (occludin, claudin-5, ZO proteins) [48] and increase metalloproteinase-9 (MMP-9) levels, which damages the BBB. Virus-infected leukocytes expressing ACE2 can transmit SARS-CoV-2 to the brain through the vascular system, meninges, and choroid plexus through the ‘Trojan horse’ mechanism- by recruiting to the brains of neutrophilia-infected people (this mechanism was demonstrated by the authors in a study on human and mouse cells in the case of encephalitis caused by West Nile virus) [49].

## 6. Autoimmunity of the Nervous System

The presence of molecular mimicry, an autoimmune response associated with the production of autoantibodies that cross-react with brain tissue, may initiate its damage [50,51], while persistently elevated values of neuroimmune biomarkers, such as glial fibrillary acidic protein (GFAP), neurofilament light chain (NFL), C-C motif chemokine ligand 2 (CCL2), monocyte chemoattractant protein-1 (MCP-1), and galactin 6, confirm immune activation in the central nervous system [52].

Elevated levels of these molecules were detected in the serum and CSF of patients with long-term COVID. In preclinical findings, Gutman et al. validated increased NFL levels more frequently in patients with cognitive impairment and fatigue in long COVID [53]. Synaptic function biomarkers SNAP-25 and neurogranin measured in CSF were decreased [27]. Disrupted production of neurotransmitters such as serotonin (tryptophan Trp degradation leads to decreased serotonin levels) contributes to the development or exacerbation of psychiatric disorders, low motivation, memory impairment and executive dysfunction [54,55,56].

Dysregulation of immune-inflammatory pathways affects hippocampal function and synaptic plasticity, playing a significant role in the development of neurological and neuropsychiatric symptoms [57].

Viral proteins—spike (S) and nucleocapsid (N) proteins—exhibit sequence homology with neuronal antigens. This structural similarity may lead to cross-reactivity of CD8+ T cells and the production of autoantibodies against myelin oligodendrocyte glycoprotein (MOG) and the N-methyl-D-aspartate receptor (NMDAR) on the surface of central nervous system cells [58,59,60].

## 7. Cognitive Dysfunction in Patients After COVID-19 Infection

The cohorts of numerous retrospective studies have shown that one-third of patients within 6 months after SARS-CoV-2 infection developed neurological or psychiatric symptoms [39,61]. Cognitive dysfunction was found in more than 60% of all subjects [61].

However, in a cohort study of 1438 participants treated for COVID-19, Liu et al. found a 12.45% prevalence of cognitive impairment in survivors 12 months after hospital discharge. Severe COVID-19 was associated with a higher risk of early and progressive cognitive impairment. The authors emphasize that COVID-19 survival was associated with an increased risk of long-term cognitive decline, highlighting the importance of immediate actions to address this challenge [62].

Elevated levels of IL-6 and TNF-α in the cerebrospinal fluid (CSF) triggered stress response mechanisms disrupting synaptic plasticity and hippocampal neurogenesis [63]. Brain dysfunction and neuronal damage are caused by either direct cytolysis or secondary inflammatory responses as a result of viral infection. Regardless of how SARS-CoV-2 affects the brain, by primary or secondary routes, the neurological complications of COVID-19 may be related to the invasive effects of SARS-CoV-2 on brain tissue [64].

Cognitive impairments in domains such as memory, attention, executive functions, language, visuospatial functions, and information processing speed should be assessed with standardized neuropsychological tests such as the Montreal Cognitive Assessment (MoCA), Mini-Mental State Examination (MMSE), and Symbol Digit Modalities Test (SDMT). The results of these tests have confirmed the occurrence of impaired attention, executive functions, verbal fluency, and working memory in patients with long COVID [65,66].

Cognitive impairment is a common and debilitating symptom of long COVID-19, often manifesting as post-infectious fatigue syndrome, as demonstrated in a prospective study carried out by Graham et al., in which half of the patients had abnormal neurological examination results, and the most frequently reported symptoms were impaired short-term memory and attention [10]. Fontes-Dantas and co-authors proposed that the spike protein is associated with long COVID-19-related brain dysfunction, independent of SARS-CoV-2 replication in the brain; the spike protein or its S1 fragments can be transported by inflammatory cells to the central nervous system, where it crosses the blood–brain barrier (BBB) and binds to Toll-like 4 receptors, stimulating the production of pro-inflammatory mediators, exacerbating and perpetuating inflammation, and affecting memory-related areas [67].

The presence of abnormal magnetic resonance imaging (MRI) findings in patients with COVID-19 and the detection of SARS-CoV-2 RNA in the cerebrospinal fluid confirms the neuroinvasive potential of the virus [68,69,70], leading to damage and loss of brain neurons and endothelial cells. This contributes to the occurrence of neurological symptoms in patients.

Elevated levels of IL-6 and TNF-α in the cerebrospinal fluid (CSF) can trigger stress response mechanisms disrupting synaptic plasticity and hippocampal neurogenesis [71].

Regardless of how SARS-CoV-2 affects the brain, by primary or secondary routes, the neurological complications of COVID-19 may be related to the invasive effects of SARS-CoV-2 on brain tissue [72,73,74].

### 7.1. Virus Invasion into the Central Nervous System

There are two potential main routes for the virus to enter the CNS: neuroinvasion and damage to the BBB [75]. SARS-CoV-2 RNA has not only been detected in the olfactory mucosa and olfactory bulb but also in various branches of the trigeminal nerve, where it enters through the ACE receptor [76].

Potential entry virus into the CNS involves hematogenous spread and retrograde neuronal transmission via olfactory neurons in the cribriform plate.

Each olfactory receptor neuron projects dendrites into the nasal cavity and extends its axons basolaterally through the cribriform plate to the olfactory bulb in the brain [77,78,79]. Through this pathway, SARS-CoV-2 in the nasal endothelium can attach to motor proteins along sensory and olfactory nerves to reach the brain [80].

The olfactory mucosa of mice and humans has been shown to express two key proteins involved in SARS-CoV-2 entry: ACE 2 and TMPRSS 2 [81,82]. In a post-mortem study, immunostaining confirmed the expression of NRP-1 in the superficial layer of human respiratory and olfactory epithelium [83]. Intranasal inoculation of mice with SARS-CoV-2 showed that the virus entered the brain through the olfactory nerve and then spread to brain regions such as the thalamus and brainstem [84]. Therefore, after inhaling SARS-CoV-2 into the nasal cavity, the virus may enter the central nervous system along the recurrent axons of the olfactory nerve through ACE2 and TMPRSS2 receptors on the olfactory mucosa, causing neuronal necrosis and dysfunction, and thus, cognitive impairment due to CNS damage [63].

### 7.2. The Effect of Inflammation on the Central Nervous System

Persistent systemic inflammation during COVID-19 infection has been shown to be associated with subsequent cognitive decline, relative to the observed hippocampal atrophy [85,86]. In addition, patients with COVID-19 had impaired coagulation, elevated D-dimer levels, thrombocytopenia, a hypercoagulable state and an increased risk of ischaemic stroke.

Potential biomarker findings are complemented by advanced neurodiagnostic techniques, including magnetic resonance imaging (MRI) and positron emission tomography (PET), which have revealed hypometabolism in the frontal and parietal cortex, decreased cortical thickness, and white matter changes in patients with cognitive impairment, while neuroinflammation and vascular damage have been demonstrated in PET scans [87,88,89,90,91,92]. Imaging studies indicate disturbances in the permeability of the blood–brain barrier, which confirms the neurovascular model of COVID-19-induced damage to the central nervous system [93].

The efficiency of complex cognitive functions depends on many cortical and subcortical brain structures and the neuronal connections between them. Structures such as the frontal and temporal lobes, hippocampus, thalamus and areas of the association cortex are mainly responsible for the most important cognitive functions. Damage and ischaemia within these structures are associated with decreased cognitive functions. Hypoxia, encephalitis and stroke cause structural and functional damage to the CNS, which then results in long-term or even permanent impairment of neurocognitive function [94].

## 8. Long-Term Effects of COVID-19

COVID-19 infection contributes to cerebral complications (cognitive and neuropsychiatric disorders) Table 1. Concentration difficulties, insomnia, and psychotic disorders such as hallucinations, delusions and behavioural changes, as well as affective disorders, including suicidality, are the most commonly reported complications [95]. About 30% of patients with severe SARS-CoV-2 developed neurological and psychiatric disorders [61,96]. Some of these patients developed autoimmune-associated anti-NMDA receptor encephalitis. Patients who have cerebrospinal fluid antibodies against the NMDA receptor might develop working memory deficits, attentional deficits, confusion, agitation, and acute psychosis symptoms such as auditory hallucinations, catatonia, and speech dysfunction. This indicates that clinicians should pay attention to the possibility of developing autoimmune encephalitis in patients with severe COVID-19 [96].

Some reports indicate that neuropsychiatric symptoms such as impaired immediate verbal memory and learning, deferred verbal memory, impaired verbal fluency, impaired working memory, anxiety, and depression persisted for at least one year in patients who recovered from COVID-19 [19,97]. Once severe respiratory symptoms have resolved, patients are at increased risk of long-term neuropsychiatric conditions such as depression, obsessive-compulsive disorder, and neurodegenerative diseases such as Alzheimer’s and Parkinson’s disease [98]. The neurobiology of these symptoms is not fully understood, but it is postulated that an excessive cytokine response plays an important role. Increasing IL-1β and IL-6 expression in microglia can induce nervous system inflammation and neuronal damage, causing neurotransmission abnormalities and functional changes in the brain. High levels of IL-1β in the cerebrospinal fluid (CSF) in COVID-19 patients damage the blood–brain barrier (BBB), allowing cytokines and immune cells to enter the brain [99].

The effects of SARS-CoV-2 infection can manifest many years after the infection, so it is essential to monitor patients who had COVID-19 and adopt measures to mitigate or prevent cognitive impairment associated with the disease.

The long-term effects of COVID-19 are attributed to persistent inflammation of the nervous system, especially immune system dysregulation, and pro-inflammatory cytokines.

### 8.1. Long COVID Symptoms

The most common symptom is anxiety, which persists in approximately one-third of patients for more than 6 months after illness onset. This fatigue can be mental or emotional, and may result in weakened muscle strength [100,101]. The consequences of long-term fatigue are serious for both the patient and those around them, sometimes preventing normal functioning in daily activities and relationships, causing emotional distress. Many studies have demonstrated a link between anxiety and depression and the severity of COVID-19, especially in women [102]. Currently, we do not have tools that would clearly show whether depressive anxiety disorders are a complication of cognitive disorders in long COVID or contribute to their occurrence.

About 30% of COVID-19 survivors may experience anxiety, depression, and posttraumatic stress that persist for months after recovery, leading to a need for psychological intervention and psychiatric treatment [103,104]. Common variables associated with depression included female gender, previous psychiatric history, and psychological stress [105]. Inflammation may contribute to the occurrence of psychiatric disorders, especially depression; this may be related to increased levels of pro-inflammatory cytokines and acute phase proteins in the chronic state [106]. Patient with depression have high levels of pro-inflammatory cytokines (TNF-α, IL-1β, IL-6), C-reactive protein, α-1-acid glycoprotein, α-1-antichymotrypsin, and haptoglobin in their blood, as well as increased expression of adhesion molecules and chemokines [107,108]. The kynurenine pathway is activated by reducing tryptophan and quinolic acid production, affecting neurotransmitters and BDNF production [109]. In the microglia, on the other hand, IL-8 is produced and, through positive feedback, maintains inflammation [110].

Benedetti et al. published data from a magnetic resonance imaging (MRI) study showing that the severity of depressive disorders is associated with a decrease in grey matter volume in the anterior cingulate cortex [103]. A significant association was observed between the severity of inflammation during acute COVID-19, brain structure and function, and the clinical diagnosis of depression and posttraumatic stress disorder in survivors [103]. These results suggest that increased cytokine levels affect neurotransmitters such as monoamines and glutamate in the amygdala, insula, and anterior cingulate cortex, which are areas associated with anxiety [107].

**Table 1:** Neuropsychiatric complications of long COVID.

			Type of Symptoms	References
Brain complications	Cognitive and neuropsychiatric disorders	cognitive impairment (confirmed by neuropsychological tests):	- memory, especially short-term memory - attention - executive functions - visuospatial function - speed of information processing	[10,65,66]
		Fatigue:	- mental - emotional - muscle weakness	[100,101]
		subjective feeling of “brain fog”:	- decreased psychomotor performance - lack of mental clarity - difficulty concentrating - impairment of function - executive	[19,97]
		psychotic disorders	- hallucinations - delusions - changes in behaviour - suicidal thoughts	[95]
		anxiety		[102]
		depression		[102]
		posttraumatic stress		[103,104]
		obsessive-compulsive disorder		[98]
		neurodegenerative diseases	(Alzheimer’s disease, Parkinson’s disease)	[98]

### 8.2. Long COVID Neuropsychiatric Disorders

The most clinically significant symptoms of the neuroprogressive process are cognitive decline and affective syndromes. “Brain fog” is defined as reduced psychomotor performance, lack of mental clarity, disorientation, difficulty thinking or concentrating and impaired executive function [111,112,113]. It has occurred more frequently in women who required hospitalization for infections, were in the intensive care unit, and had breathing difficulties. Depressive disorders are associated with impairments in cognition, episodic memory, executive function, processing speed, visuospatial memory, attention, and working memory as neuropsychiatric symptoms [114], although according to other authors, it occurs regardless of age and COVID-19 exacerbation [115].

Cognitive deficits have been observed in 20–35% of patients after the resolution of the acute form of COVID-19, impairing concentration, short-term memory, attention, frontal and executive functions, depression, or anxiety disorders lasting more than 3 months [116,117,118,119,120]. In a study by Graham and Clark, people with COVID-19 who were not hospitalized and did not have prolonged brain fog experienced pronounced and persistent brain fog and fatigue that impacted their cognitive abilities and quality of life [121]. At the individual level, deficits are usually mild but may have significant functional significance, especially in patients with severe COVID-19, suggesting that this infection may accelerate pre-existing neurodegenerative, inflammatory, or cerebrovascular vulnerabilities [122].

### 8.3. Long COVID in Children and Adolescents

Long COVID in paediatrics presents with heterogeneous severity and duration [123]. In a meta-analysis by Lopez, Wegman, et al., the prevalence of long COVID-19 in children and adolescents was 25.24%. The most common clinical symptoms included mood disorders (16.50%), fatigue (9.66%), sleep disturbances (8.42%), headache (7.84%), and respiratory symptoms (7.62%). Risk factors associated with long COVID-19 included the following: older age, female gender, severe COVID-19, overweight/obesity, concomitant allergic diseases, and other long-term comorbidities [124].

Currently, long-term observational studies evaluating neurodevelopmental outcomes in children after infection with nervous system viruses are limited. The highest burden of diseases associated with CNS infections is in low- and middle-income countries. Longitudinal studies are important in tracking the impact of SARS-CoV-2 infection on the CNS so that we can better understand and take effective intervention [125].

## 9. Predictors of “Brain Fog”

Depression is the strongest predictor of brain fog, and it has been postulated that neuroinflammation may be responsible for both “brain fog” and depressive disorders. During a prolonged immune response, in which inflammatory cells and mediators cross the BBB, neuroinflammation develops with elevated plasma TNF-α levels, which has been associated with cognitive deficits several months after recovery from acute COVID-19 infection [126,127]. Increased levels of IL-6, CRP, soluble IL-6 receptor, and TNF-α are responsible for persistent low-grade inflammation leading to the development of post-viral neurocognitive syndromes, which include cognitive dysfunctions, mood disorders, and fatigue [128]. NFL and GFAP are responsible for persistent neurodegeneration, indicating damage to axons and astrocyte cells [129].

It is estimated that over 10 million patients suffer from neurocognitive and neuropsychiatric dysfunction due to the neurological manifestation of COVID-19, leading to a decline in the quality of life and an inability to return to previous functioning levels. Long-term follow-up is necessary, as autoimmune effects may become apparent much later [130].

## 10. Neurodegeneration in the Course of COVID-19

Viral neurotropic infections can cause neuronal dysfunction by promoting chronic inflammation, induction of oxidative stress in cells, impairment of mitophagy and mitochondria, alteration of neurotransmitter systems, or induction of misfolded and aggregated pathological proteins associated with neurodegenerative diseases. This is the cause of multidimensional brain damage leading to specific neuronal and brain disorders [131].

The neuropsychiatric sequelae of COVID-19 resemble a neuroprogressive process, in which acute inflammatory and vascular damage can lead to permanent CNS dysfunction, depressive and anxiety disorders, and cognitive decline. The concept of downstaging as a therapeutic option aimed at reversing or attenuating disease progression by influencing impaired neuroplasticity and immune dysregulation would disrupt the possibility of perpetuating the cognitive-affective syndrome [132,133]. This is in line with contemporary translational neuropsychiatry, where the therapeutic goal is to slow the progression of the stage, preserve cognitive functions, prevent the consolidation of depressive and anxiety phenotypes after COVID-19, and treat already existing symptoms [17,133].

Long-term consequences of neuroinflammation include persistent deficits in attention, memory, and executive functions, and may increase the vulnerability and progression of neurodegenerative diseases such as Parkinson’s and Alzheimer’s disease. The incidence of dementia increases with age, often with a prolonged subclinical latency period. Survivors of SARS-CoV-2 infection are among the population most at risk for accelerated or de novo neurodegenerative disease [134].

Recent studies indicate that SARS-CoV-2 infection may induce neurodegenerative changes in Parkinson’s disease (PD) by accelerating ageing and accumulating abnormal proteins in the brain. In contrast, the basis of neurodegeneration in Alzheimer’s disease (AD) is an amyloid-stimulated interferon (IFN) response resulting in inflammation of the central nervous system [135]. As a result of SARS-CoV-2 infection, the neurodegeneration process is associated with microglia activation and mitochondrial dysfunction development, and the increased levels of α-synuclein, total Tau, phosphorylated (pTau), β-amyloid (Aβ42) in serum, and CSF may accelerate these processes [136].

Kim and Yang demonstrated that SARS-CoV-2 infection of DA neurons triggers an inflammatory response and cellular senescence. Midbrain dopamine (DA) neurons derived from human pluripotent stem cells (hPSCs) are selectively sensitive and susceptible to SARS-CoV-2 infection. The researchers observed a reduced number of neuromelanin- and tyrosine hydroxylase (TH)-positive DA neurons and fibres in a cohort of patients with severe COVID-19. These findings demonstrate that hPSC-derived DA neurons are sensitive to SARS-CoV-2. This represents a potential group of patients in whom the use of neuroprotective drugs may alleviate symptoms or halt the neurodegeneration process [137].

Moreover, viral replication can also hijack the protein synthesis machinery in neurons, promote the response to misfolded proteins, and disrupt proteostasis, thus leading to the accumulation of misfolded proteins and subsequent aggregation [43,138,139].

## 11. Neurodegenerative Diseases—Alzheimer’s Disease

Viral infection is considered an aetiological factor in Alzheimer’s disease [140]. However, it is not known whether SARS-CoV-2 virus infection is associated with an increased risk of the disease. The long-term neurological complications of virus infection are partially caused by inflammation, which plays a central role in the neuropathology of Alzheimer’s disease [141]. It is not clear whether COVID-19 can induce de novo Alzheimer’s disease or accelerate its onset. Nonetheless, coronaviruses have been detected in the CNS of patients with Parkinson’s (PD) and Alzheimer’s disease (AD) [135].

After binding to ACE2 receptors, which are widely distributed in the brain and can be found in dopamine neurons of the striatum, SARS-CoV-2 can infect the brain, including even the brainstem [142,143,144]. COVID-19 may increase the risk of developing diseases as a distant complication of the infection. Therefore, it can be speculated that survivors represent a potential population at higher risk of developing neurodegenerative diseases as a result of silent viral infection in the brain.

The severe complications of SARS-CoV-2 infection are most often associated with a “cytokine storm” resulting from inflammation and increased levels of pro-inflammatory cytokines. In patients with AD, this may act synergistically with the amyloid-stimulated Interferon-1 (IFN) response, leading to a so-called “perfect storm”. In some patients with undiagnosed AD, SARS-CoV-2 infection may result in accelerated disease initiation and earlier onset of AD symptoms [145].

## 12. COVID and Parkinson’s Disease

In patients with Parkinson’s disease, anti-coronavirus antibodies were found in the cerebrospinal fluid (PMR) more than two decades ago [146]. Pre-entry of coronaviruses into the brain through the nasal cavity causes olfactory abnormalities, a premotor feature of Parkinson’s disease, while alpha-synuclein (aSyn), a major protein component of Lewy bodies found in the brain of PD patients, is deposited in the olfactory bulb [147]. Lippi et al. investigated the role of SARS-CoV-2 in the development of neurodegenerative diseases [43]. They highlighted cellular pathways in the course of viral infection, such as proteostasis (protein homeostasis). These pathways maintain dynamic equilibrium and activate stress response mechanisms, which also appear to be involved in neurodegeneration [43,148]. In vitro studies have shown that alterations in proteostasis can lead to the accumulation of toxic insoluble proteins [149]. SARS-CoV2 infection can lead to PD neurodegeneration by accelerating brain cell ageing [142].

Conversely, the neuropathology of Parkinson’s disease itself may trigger a protective effect against COVID-19 infection. The infection develops as SARS-CoV-2 binds to ACE2 receptors, which are also found in striatal dopamine neurons [143]. PD patients present a reduced number of black matter dopamine neurons as a result of neurodegeneration with Lewy bodies (eosinophilic inclusions present in the cytoplasm of nerve cells, composed mainly of α-synuclein protein). In addition, α-synuclein inhibits viral neuroinvasion, providing protection against pro-inflammatory response [43,150].

Further studies are needed to better elucidate the links between clinical status and COVID-19 severity, systemic inflammatory response with cytokine levels and virus detection in the cerebrospinal fluid of PD patients.

After entering the central nervous system (CNS), SARS-CoV-2 invades and replicates within neurons, leading to their death. Infection of certain brain regions, particularly the brainstem, can lead to cardiorespiratory dysfunction and even death. Viral invasion and neuronal death activate astrocytes and microglia, triggering an inflammatory response. This, combined with the intense release of cytokines during an intense cytokine storm, can increase the permeability of the BBB, thus facilitating SARS-CoV-2’s entry into the CNS.

In sum, long-term complications following COVID-19 infection may be associated with neurodegenerative diseases [138,151].

## 13. Clinical Implications

Using data extracted from the electronic medical records of two large cohorts, Peluso and Deeks [152] identified four reproducible long COVID subphenotypes that were associated with distinct patient demographics, comorbidities prior to SARS-CoV-2 infection, and the severity of the acute phase of infection.

In the first subphenotype, constituting 33.75%, patients, who were predominantly male, had cardiovascular disease, renal failure, anaemia, and water-electrolyte disorders. In this group, acute COVID-19 was associated with the highest rates of hospitalization (61.15%) and mechanical ventilation (4.81%). The second subphenotype mainly concerned women with respiratory diseases, sleep disorders, anxiety and symptoms such as headache and chest pain. Patients predominantly women with musculoskeletal and nervous system complications such as musculoskeletal pain, headaches, and sleep and wakefulness disorders constituted the third subphenotype. The mildest clinical manifestations were observed in the group of patients in the fourth subphenotype of 10.14%, with a predominance of women and musculoskeletal and nervous system complications such as musculoskeletal pain, headache, and sleep and wakefulness disorders.

Patients with subphenotypes 1 and 2 were more likely to be hospitalized in intensive care units and required artificial ventilation in acute COVID than patients with subphenotypes 3 and 4.

Other authors have identified a lower overall burden of underlying diseases alongside a slightly higher incidence of gastrointestinal diseases, such as hematemesis, gastroduodenal disorders, and gastrointestinal cancers, based on the number of symptoms. Currently, many endotypes exhibit overlapping characteristics, making their application in practice challenging. It would be helpful to identify objectively defined phenotypes that can be readily applied in clinical settings.

Long COVID is not limited to any single organ system and has documented respiratory, cardiovascular, neuropsychiatric, and endocrine complications [153,154,155].

Common limitations reported in these meta-analyses include population heterogeneity, a lack of consensus regarding the definition and diagnosis of the post-COVID-19 state, and diverse geographic locations.

We noted that studies involving meta-analyses had a high probability of bias, including a lack of standardized definitions, incorrect classification, recall, selection, loss of observation, or lack of response. In addition, the included meta-analysis studies have limitations inherited in all observational studies, such as a high level of heterogeneity and error due to residual and unmeasured confounding factors. This may have been due to differences in study design, different environments, different study groups, follow-up time, methods of recognizing symptoms, different nomenclature, limited data on the stratification of comorbidities, and previous vaccination against COVID-19.

## 14. Conclusions

Although the COVID-19 pandemic has passed, complications resulting from COVID-19 continue to impact patients’ health. Comprehensive assessment of possible neuropsychiatric disorders, particularly a vulnerability to cognitive decline and dementia in the future, will be an important diagnostic and therapeutic challenge, as well as an important aspect of improving patients’ quality of life. It is still not fully understood whether SARS-CoV-2 causes de novo neurodegenerative diseases or whether it accelerates or exacerbates pre-existing neurodegenerative diseases and cognitive impairment. This is a social issue that cannot be ignored. Analyses using independent, newer patient cohorts and prospective studies are necessary to further validate the observed inflammatory and degenerative phenotypes. It is important to monitor patients post-infection, and personalize treatment based on their phenotype and immunological biomarkers. Potential prognostic, diagnostic, and treatment response monitoring tools include immune biomarkers such as interleukins IL-6 and cytokine TNF-α, structural proteins GFAP and NFL, neurotransmitters serotonin and dopamine, and CNS dysfunction indicators, which enable disease progression tracking and treatment response, rehabilitation, and cognitive therapy assessment. However, their clinical value in long COVID monitoring has not yet been clearly established. Therapeutic strategies targeting inflammatory mediators, particularly the IL-6 pathway (tocilizumab) and TNF inhibitors (currently in clinical trials), may demonstrate benefits in long COVID. A promising therapeutic approach to inhibiting neurodegeneration involves inhibiting transcription factor NF-kB, which enhances glial cytokine production. Future research should focus on determining and monitoring immunological biomarkers, radiological studies, and neuropsychological testing to enable the early identification of long COVID patients with cognitive impairment and reduce the risk of developing neurodegenerative diseases.

In clinical trials, promising results have been demonstrated using therapy based on mitochondrial miRNA antagonists to increase the expression of specific proteins that are downregulated, or to decrease the expression of the proteins they abnormally induce [156].

Therefore, a deeper understanding of the mechanisms underlying SARS-CoV-2 infection is essential for the development of more effective preventive and therapeutic strategies to protect at-risk groups.

## Data Availability

No new data were created or analysed in this study. Data sharing is not applicable to this article.

## References

[1] Tyrrell D.A.J., Bynoe M.L. (1966). Cultivation of viruses from a high proportion of patients with colds. Lancet.

[2] Zhou P., Yang X.-L., Wang X.-G., Hu B., Zhang L., Zhang W., Si H.-R., Zhu Y., Li B., Huang C.-L. (2020). A pneumonia outbreak associated with a new coronavirus of probable bat origin. Nature.

[3] Uraki R., Korber B., Diamond M.S., Kawaoka Y. (2026). SARS-CoV-2 variants: Biology, pathogenicity, immunity, and control. Nat. Rev. Microbiol..

[4] Number of COVID-19 Deaths Reported to WHO. WHO COVID-19 Dashboard. https://data.who.int/dashboards/covid19/deaths.

[5] McVoy M.A., Kummarapurugu A.B. (2025). The Role of ACE2 in SARS-CoV-2 Infection, Pathogenesis, and Antiviral Interventions. J. Med. Virol..

[6] Mohanty A., Agnihotri S., Mehta A., Rawal S. (2021). COVID-19 and cancer: Sailing through the tides. Pathol.—Res. Pract..

[7] Channappanavar R., Perlman S. (2017). Pathogenic human coronavirus infections: Causes and consequences of cytokine storm and immunopathology. Semin. Immunopathol..

[8] Lyra E Silva N.M., Barros-Aragão F.G.Q., De Felice F.G., Ferreira S.T. (2022). Inflammation at the crossroads of COVID-19, cognitive deficits and depression. Neuropharmacology.

[9] A Clinical Case Definition of Post COVID-19 Condition by a Delphi Consensus, 6 October 2021. https://www.who.int/publications/i/item/WHO-2019-nCoV-Post_COVID-19_condition-Clinical_case_definition-2021.1.

[10] Cáceres E., Divani A.A., Viñan-Garces A.E., Olivella-Gomez J., Quintero-Altare A., Pérez S., Reyes L.F., Sasso N., Biller J. (2025). Tackling persistent neurological symptoms in patients following acute COVID-19 infection: An update of the literature. Expert Rev. Neurother..

[11] Woodrow M., Carey C., Ziauddeen N., Thomas R., Akrami A., Lutje V., Greenwood D.C., Alwan N.A. (2023). Systematic Review of the Prevalence of Long COVID. Open Forum Infect. Dis..

[12] Davis H.E., McCorkell L., Vogel J.M., Topol E.J. (2023). Long COVID: Major findings, mechanisms and recommendations. Nat. Rev. Microbiol..

[13] Spedding M., Aerts J., Alexander S., Bellozzi Woestelandt A.-G., Chiricozzi E., Henriques A., Lledo P.-M., Loeffler J.-P., Perera R., Platt F.M. (2026). Links between COVID-19, long COVID, and neurodegeneration: The role of glycosphingolipids. Pharmacol. Rev..

[14] National Center for Health Statistics Long COVID—Household Pulse Survey. https://www.cdc.gov/nchs/covid19/pulse/long-covid.htm.

[15] Fernández-Castañeda A., Lu P., Geraghty A.C., Song E., Lee M.-H., Wood J., O’Dea M.R., Dutton S., Shamardani K., Nwangwu K. (2022). Mild respiratory COVID can cause multi-lineage neural cell and myelin dysregulation. Cell.

[16] Boechat J.L., Chora I., Morais A., Delgado L. (2021). The immune response to SARS-CoV-2 and COVID-19 immunopathology—Current perspectives. Pulmonology.

[17] Hein Z.M., Thazin, Kumar S., Che Ramli M.D., Che Mohd Nassir C.M.N. (2025). Immunomodulatory Mechanisms Underlying Neurological Manifestations in Long COVID: Implications for Immune-Mediated Neurodegeneration. Int. J. Mol. Sci..

[18] Wang P., Jin L., Zhang M., Wu Y., Duan Z., Guo Y., Wang C., Guo Y., Chen W., Liao Z. (2024). Blood–brain barrier injury and neuroinflammation induced by SARS-CoV-2 in a lung–brain microphysiological system. Nat. Biomed. Eng..

[19] Soung A.L., Vanderheiden A., Nordvig A.S., Sissoko C.A., Canoll P., Mariani M.B., Jiang X., Bricker T., Rosoklija G.B., Arango V. (2022). COVID-19 induces CNS cytokine expression and loss of hippocampal neurogenesis. Brain J. Neurol..

[20] Sun Y., Wong L.-Y.R., Chang T.L. (2026). ACE2-independent entry factors for SARS-CoV-2 infection and immune activation. mBio.

[21] Singh A.K., Bhushan B., Maurya A., Mishra G., Singh S.K., Awasthi R. (2020). Novel coronavirus disease 2019 (COVID-19) and neurodegenerative disorders. Dermatol. Ther..

[22] Varga Z., Flammer A.J., Steiger P., Haberecker M., Andermatt R., Zinkernagel A.S., Mehra M.R., Schuepbach R.A., Ruschitzka F., Moch H. (2020). Endothelial cell infection and endotheliitis in COVID-19. Lancet.

[23] Paniz-Mondolfi A., Bryce C., Grimes Z., Gordon R.E., Reidy J., Lednicky J., Sordillo E.M., Fowkes M. (2020). Central nervous system involvement by severe acute respiratory syndrome coronavirus-2 (SARS-CoV-2). J. Med. Virol..

[24] Monje M., Iwasaki A. (2022). The neurobiology of long COVID. Neuron.

[25] Brudno J.N., Kochenderfer J.N. (2016). Toxicities of chimeric antigen receptor T cells: Recognition and management. Blood.

[26] du Plessis M., Fourie C., Riedemann J., de Villiers W.J.S., Engelbrecht A.M. (2022). Cancer and COVID-19: Collectively catastrophic. Cytokine Growth Factor Rev..

[27] Day G.S., Yarbrough M.Y., Körtvelyessy P., Prüss H., Bucelli R.C., Fritzler M.J., Mason W., Tang-Wai D.F., Steriade C., Hébert J. (2021). Prospective Quantification of CSF Biomarkers in Antibody-Mediated Encephalitis. Neurology.

[28] Chu H., Zhou J., Wong B.H.-Y., Li C., Chan J.F.-W., Cheng Z.-S., Yang D., Wang D., Lee A.C.-Y., Li C. (2016). Middle East Respiratory Syndrome Coronavirus Efficiently Infects Human Primary T Lymphocytes and Activates the Extrinsic and Intrinsic Apoptosis Pathways. J. Infect. Dis..

[29] Law H.K.W., Cheung C.Y., Ng H.Y., Sia S.F., Chan Y.O., Luk W., Nicholls J.M., Peiris J.S.M., Lau Y.L. (2005). Chemokine up-regulation in SARS-coronavirus–infected, monocyte-derived human dendritic cells. Blood.

[30] Mehta P., McAuley D.F., Brown M., Sanchez E., Tattersall R.S., Manson J.J. (2020). COVID-19: Consider cytokine storm syndromes and immunosuppression. Lancet.

[31] Huang K., Su I., Theron M., Wu Y., Lai S., Liu C., Lei H. (2005). An interferon-γ-related cytokine storm in SARS patients. J. Med. Virol..

[32] Zhao C., Zhao W. (2020). NLRP3 Inflammasome—A Key Player in Antiviral Responses. Front. Immunol..

[33] Urwanisch L., Luciano M., Horejs-Hoeck J. (2021). The NLRP3 Inflammasome and Its Role in the Pathogenicity of Leukemia. Int. J. Mol. Sci..

[34] Zhang W.-J., Chen S.-J., Zhou S.-C., Wu S.-Z., Wang H. (2021). Inflammasomes and Fibrosis. Front. Immunol..

[35] Verdecchia P., Cavallini C., Spanevello A., Angeli F. (2020). The pivotal link between ACE2 deficiency and SARS-CoV-2 infection. Eur. J. Intern. Med..

[36] Fu Y., Cheng Y., Wu Y. (2020). Understanding SARS-CoV-2-Mediated Inflammatory Responses: From Mechanisms to Potential Therapeutic Tools. Virol. Sin..

[37] Albornoz E.A., Amarilla A.A., Modhiran N., Parker S., Li X.X., Wijesundara D.K., Aguado J., Zamora A.P., McMillan C.L.D., Liang B. (2023). SARS-CoV-2 drives NLRP3 inflammasome activation in human microglia through spike protein. Mol. Psychiatry.

[38] Gadhave D.G., Sugandhi V.V., Jha S.K., Nangare S.N., Gupta G., Singh S.K., Dua K., Cho H., Hansbro P.M., Paudel K.R. (2024). Neurodegenerative disorders: Mechanisms of degeneration and therapeutic approaches with their clinical relevance. Ageing Res. Rev..

[39] Chen Y., Yang W., Chen F., Cui L. (2022). COVID-19 and cognitive impairment: Neuroinvasive and blood–brain barrier dysfunction. J. Neuroinflamm..

[40] Hamming I., Timens W., Bulthuis M.L.C., Lely A.T., Navis G.J., van Goor H. (2004). Tissue distribution of ACE2 protein, the functional receptor for SARS coronavirus. A first step in understanding SARS pathogenesis. J. Pathol..

[41] Kanwar D., Baig A., Wasay M. (2020). Neurological manifestations of COVID-19. J. Pak. Med. Assoc..

[42] Fotuhi M., Mian A., Meysami S., Raji C.A. (2020). Neurobiology of COVID-19. J. Alzheimers Dis. JAD.

[43] Lippi A., Domingues R., Setz C., Outeiro T.F., Krisko A. (2020). SARS-CoV-2: At the Crossroad Between Aging and Neurodegeneration. Mov. Disord. Off. J. Mov. Disord. Soc..

[44] Garber C., Soung A., Vollmer L.L., Kanmogne M., Last A., Brown J., Klein R.S. (2019). T cells promote microglia-mediated synaptic elimination and cognitive dysfunction during recovery from neuropathogenic flaviviruses. Nat. Neurosci..

[45] Castro Dias M., Mapunda J.A., Vladymyrov M., Engelhardt B. (2019). Structure and Junctional Complexes of Endothelial, Epithelial and Glial Brain Barriers. Int. J. Mol. Sci..

[46] Suprewicz Ł., Fiedoruk K., Czarnowska A., Sadowski M., Strzelecka A., Galie P.A., Janmey P.A., Kułakowska A., Bucki R. (2023). Blood-brain barrier function in response to SARS-CoV-2 and its spike protein. Neurol. Neurochir. Pol..

[47] Yamada S., Hashita T., Yanagida S., Sato H., Yasuhiko Y., Okabe K., Noda T., Nishida M., Matsunaga T., Kanda Y. (2024). SARS-CoV-2 causes dysfunction in human iPSC-derived brain microvascular endothelial cells potentially by modulating the Wnt signaling pathway. Fluids Barriers CNS.

[48] Yang F., Zhao K., Zhang X., Zhang J., Xu B. (2016). ATP Induces Disruption of Tight Junction Proteins via IL-1 Beta-Dependent MMP-9 Activation of Human Blood-Brain Barrier In Vitro. Neural Plast..

[49] Paul A.M., Acharya D., Duty L., Thompson E.A., Le L., Stokic D.S., Leis A.A., Bai F. (2017). Osteopontin facilitates West Nile virus neuroinvasion via neutrophil “Trojan horse” transport. Sci. Rep..

[50] Lavi Y., Vojdani A., Halpert G., Sharif K., Ostrinski Y., Zyskind I., Lattin M.T., Zimmerman J., Silverberg J.I., Rosenberg A.Z. (2023). Dysregulated Levels of Circulating Autoantibodies against Neuronal and Nervous System Autoantigens in COVID-19 Patients. Diagnostics.

[51] Dunai C., Collie C., Michael B.D. (2022). Immune-Mediated Mechanisms of COVID-19 Neuropathology. Front. Neurol..

[52] Park Y., Kc N., Paneque A., Cole P.D. (2024). Tau, Glial Fibrillary Acidic Protein, and Neurofilament Light Chain as Brain Protein Biomarkers in Cerebrospinal Fluid and Blood for Diagnosis of Neurobiological Diseases. Int. J. Mol. Sci..

[53] Gutman E.G., Salvio A.L., Fernandes R.A., Duarte L.A., Raposo-Vedovi J.V., Alcaraz H.F., Teixeira M.A., Passos G.F., de Medeiros K.Q.M., Hammerle M.B. (2024). Long COVID: Plasma levels of neurofilament light chain in mild COVID-19 patients with neurocognitive symptoms. Mol. Psychiatry.

[54] Almulla A.F., Thipakorn Y., Zhou B., Vojdani A., Paunova R., Maes M. (2024). The tryptophan catabolite or kynurenine pathway in long COVID disease: A systematic review and meta-analysis. Neuroscience.

[55] Cappelletti G., Carsana E.V., Lunghi G., Breviario S., Vanetti C., Di Fonzo A.B., Frattini E., Magni M., Zecchini S., Clerici M. (2023). SARS-CoV-2 hampers dopamine production in iPSC-derived dopaminergic neurons. Exp. Mol. Pathol..

[56] Wong A.C., Devason A.S., Umana I.C., Cox T.O., Dohnalová L., Litichevskiy L., Perla J., Lundgren P., Etwebi Z., Izzo L.T. (2023). Serotonin reduction in post-acute sequelae of viral infection. Cell.

[57] Frontera J.A., Simon N.M. (2022). Bridging Knowledge Gaps in the Diagnosis and Management of Neuropsychiatric Sequelae of COVID-19. JAMA Psychiatry.

[58] Shir D., Day G.S. (2022). Deciphering the contributions of neuroinflammation to neurodegeneration: Lessons from antibody-mediated encephalitis and coronavirus disease 2019. Curr. Opin. Neurol..

[59] Gutman E.G., Fernandes R.A., Raposo-Vedovi J.V., Salvio A.L., Duarte L.A., Tardim C.F., Costa V.G.C., Pereira V.C.S.R., Bahia P.R.V., da Silva M.M. (2023). Molecular Mimicry between SARS-CoV-2 Proteins and Human Self-Antigens Related with Autoimmune Central Nervous System (CNS) Disorders. Microorganisms.

[60] Cacciaguerra L., Flanagan E.P. (2024). Updates in NMOSD and MOGAD Diagnosis and Treatment: A Tale of Two Central Nervous System Autoimmune Inflammatory Disorders. Neurol. Clin..

[61] Taquet M., Geddes J.R., Husain M., Luciano S., Harrison P.J. (2021). 6-month neurological and psychiatric outcomes in 236 379 survivors of COVID-19: A retrospective cohort study using electronic health records. Lancet Psychiatry.

[62] Liu Y.-H., Chen Y., Wang Q.-H., Wang L.-R., Jiang L., Yang Y., Chen X., Li Y., Cen Y., Xu C. (2022). One-Year Trajectory of Cognitive Changes in Older Survivors of COVID-19 in Wuhan, China: A Longitudinal Cohort Study. JAMA Neurol..

[63] Tyagi K., Rai P., Gautam A., Kaur H., Kapoor S., Suttee A., Jaiswal P.K., Sharma A., Singh G., Barnwal R.P. (2023). Neurological manifestations of SARS-CoV-2: Complexity, mechanism and associated disorders. Eur. J. Med. Res..

[64] Lingor P., Demleitner A.F., Wolff A.W., Feneberg E. (2022). SARS-CoV-2 and neurodegenerative diseases: What we know and what we don’t. J. Neural Transm..

[65] Popa E., Popa A.E., Poroch M., Poroch V., Ungureanu M.I., Slanina A.M., Bacusca A., Coman E.A. (2025). The Molecular Mechanisms of Cognitive Dysfunction in Long COVID: A Narrative Review. Int. J. Mol. Sci..

[66] Damiano R.F., Caruso M.J.G., Cincoto A.V., de Almeida Rocca C.C., de Pádua Serafim A., Bacchi P., Guedes B.F., Brunoni A.R., Pan P.M., Nitrini R. (2022). Post-COVID-19 psychiatric and cognitive morbidity: Preliminary findings from a Brazilian cohort study. Gen. Hosp. Psychiatry.

[67] Fontes-Dantas F.L., Fernandes G.G., Gutman E.G., De Lima E.V., Antonio L.S., Hammerle M.B., Mota-Araujo H.P., Colodeti L.C., Araújo S.M.B., Froz G.M. (2023). SARS-CoV-2 Spike protein induces TLR4-mediated long-term cognitive dysfunction recapitulating post-COVID-19 syndrome in mice. Cell Rep..

[68] Goldstein D.S., Mina Y., Walitt B., Sullivan P., Enose-Akahata Y., Jacobson S., Nguyen M.-L., Sidenko S., Wiebold A., Smith B. (2024). Persistent Autonomic and Immunologic Abnormalities in Neurologic Post-Acute Sequelae of SARS-CoV2 Infection. Neurology.

[69] Sriwastava S., Tandon M., Podury S., Prasad A., Wen S., Guthrie G., Kakara M., Jaiswal S., Subedi R., Elkhooly M. (2021). COVID-19 and neuroinflammation: A literature review of relevant neuroimaging and CSF markers in central nervous system inflammatory disorders from SARS-COV2. J. Neurol..

[70] Deuter D., Hense K., Kunkel K., Vollmayr J., Schachinger S., Wendl C., Schicho A., Fellner C., Salzberger B., Hitzenbichler F. (2024). SARS-CoV2 evokes structural brain changes resulting in declined executive function. PLoS ONE.

[71] Devlin L., Gombolay G.Y. (2023). Cerebrospinal fluid cytokines in COVID-19: A review and meta-analysis. J. Neurol..

[72] Dai X., Cao X., Jiang Q., Wu B., Lou T., Shao Y., Hu Y., Lan Q. (2023). Neurological complications of COVID-19. QJM Mon. J. Assoc. Physicians.

[73] Ahmad S.J., Feigen C.M., Vazquez J.P., Kobets A.J., Altschul D.J. (2022). Neurological Sequelae of COVID-19. J. Integr. Neurosci..

[74] Ren A.L., Digby R.J., Needham E.J. (2021). Neurological update: COVID-19. J. Neurol..

[75] Lima M., Siokas V., Aloizou A.-M., Liampas I., Mentis A.-F.A., Tsouris Z., Papadimitriou A., Mitsias P.D., Tsatsakis A., Bogdanos D.P. (2020). Unraveling the Possible Routes of SARS-CoV-2 Invasion into the Central Nervous System. Curr. Treat. Options Neurol..

[76] Satarker S., Nampoothiri M. (2020). Involvement of the nervous system in COVID-19: The bell should toll in the brain. Life Sci..

[77] Butowt R., von Bartheld C.S. (2021). Anosmia in COVID-19: Underlying Mechanisms and Assessment of an Olfactory Route to Brain Infection. Neurosci. Rev. J. Bringing Neurobiol. Neurol. Psychiatry.

[78] Arrigoni A., Previtali M., Bosticardo S., Pezzetti G., Poloni S., Capelli S., Napolitano A., Remuzzi A., Zangari R., Lorini F.L. (2024). Brain microstructure and connectivity in COVID-19 patients with olfactory or cognitive impairment. NeuroImage Clin..

[79] Doty R.L. (2022). Olfactory dysfunction in COVID-19: Pathology and long-term implications for brain health. Trends Mol. Med..

[80] Tsukahara T., Brann D.H., Datta S.R. (2023). Mechanisms of SARS-CoV-2-associated anosmia. Physiol. Rev..

[81] Brann D.H., Tsukahara T., Weinreb C., Lipovsek M., Van den Berge K., Gong B., Chance R., Macaulay I.C., Chou H.-J., Fletcher R.B. (2020). Non-neuronal expression of SARS-CoV-2 entry genes in the olfactory system suggests mechanisms underlying COVID-19-associated anosmia. Sci. Adv..

[82] Essalmani R., Jain J., Susan-Resiga D., Andréo U., Evagelidis A., Derbali R.M., Huynh D.N., Dallaire F., Laporte M., Delpal A. (2022). Distinctive Roles of Furin and TMPRSS2 in SARS-CoV-2 Infectivity. J. Virol..

[83] Cantuti-Castelvetri L., Ojha R., Pedro L.D., Djannatian M., Franz J., Kuivanen S., van der Meer F., Kallio K., Kaya T., Anastasina M. (2020). Neuropilin-1 facilitates SARS-CoV-2 cell entry and infectivity. Science.

[84] Bratosiewicz-Wąsik J. (2022). Neuro-COVID-19: An insidious virus in action. Neurol. Neurochir. Pol..

[85] Díez-Cirarda M., Yus-Fuertes M., Sanchez-Sanchez R., Gonzalez-Rosa J.J., Gonzalez-Escamilla G., Gil-Martínez L., Delgado-Alonso C., Gil-Moreno M.J., Valles-Salgado M., Cano-Cano F. (2023). Hippocampal subfield abnormalities and biomarkers of pathologic brain changes: From SARS-CoV-2 acute infection to post-COVID syndrome. eBioMedicine.

[86] Bendella Z., Widmann C.N., Kindler C., Haase R., Sauer M., Heneka M.T., Radbruch A., Schmeel F.C. (2025). Longitudinal Monitoring of Brain Volume Changes After COVID-19 Infection Using Artificial Intelligence-Based MRI Volumetry. Diagnostics.

[87] Tang N., Li D., Wang X., Sun Z. (2020). Abnormal coagulation parameters are associated with poor prognosis in patients with novel coronavirus pneumonia. J. Thromb. Haemost..

[88] Sagris D., Papanikolaou A., Kvernland A., Korompoki E., Frontera J.A., Troxel A.B., Gavriatopoulou M., Milionis H., Lip G.Y.H., Michel P. (2021). COVID-19 and ischemic stroke. Eur. J. Neurol..

[89] Toniolo S., Di Lorenzo F., Scarioni M., Frederiksen K.S., Nobili F. (2021). Is the Frontal Lobe the Primary Target of SARS-CoV-2?. J. Alzheimer’s Dis. JAD.

[90] Song J., Khanduja S., Rando H., Shi W., Hazel K., Pottanat G.P., Jones E., Xu C., Hu Z., Lin D. (2024). Brain Frontal-Lobe Misery Perfusion in COVID-19 ICU Survivors: An MRI Pilot Study. Brain Sci..

[91] Zhao J., Xia F., Jiao X., Lyu X. (2024). Long COVID and its association with neurodegenerative diseases: Pathogenesis, neuroimaging, and treatment. Front. Neurol..

[92] Besteher B., Rocktäschel T., Garza A.P., Machnik M., Ballez J., Helbing D.-L., Finke K., Reuken P., Güllmar D., Gaser C. (2024). Cortical thickness alterations and systemic inflammation define long-COVID patients with cognitive impairment. Brain. Behav. Immun..

[93] Rubin L.H., Shi W., Azola A., Bhattacharyya A., Dastgheyb R.M., Wu J., Penna C.D., Parker H., Santiuste I., Ehrenspeck A. (2025). Blood-Brain barrier disruption in long COVID and cognitive correlates: A cross-sectional MRI study. Brain. Behav. Immun..

[94] Li J., Long X., Zhang Q., Fang X., Fang F., Lv X., Zhang D., Sun Y., Li N., Hu S. (2020). Emerging Evidence for Neuropsycho-Consequences of COVID-19. Curr. Neuropharmacol..

[95] Penninx B.W.J.H., Benros M.E., Klein R.S., Vinkers C.H. (2022). How COVID-19 shaped mental health: From infection to pandemic effects. Nat. Med..

[96] Vasilevska V., Guest P.C., Bernstein H.-G., Schroeter M.L., Geis C., Steiner J. (2021). Molecular mimicry of NMDA receptors may contribute to neuropsychiatric symptoms in severe COVID-19 cases. J. Neuroinflamm..

[97] Méndez R., Balanzá-Martínez V., Luperdi S.C., Estrada I., Latorre A., González-Jiménez P., Bouzas L., Yépez K., Ferrando A., Reyes S. (2022). Long-term neuropsychiatric outcomes in COVID-19 survivors: A 1-year longitudinal study. J. Intern. Med..

[98] Huang P., Zhang L.-Y., Tan Y.-Y., Chen S.-D. (2023). Links between COVID-19 and Parkinson’s disease/Alzheimer’s disease: Reciprocal impacts, medical care strategies and underlying mechanisms. Transl. Neurodegener..

[99] Greene C., Connolly R., Brennan D., Laffan A., O’Keeffe E., Zaporojan L., O’Callaghan J., Thomson B., Connolly E., Argue R. (2024). Blood–brain barrier disruption and sustained systemic inflammation in individuals with long COVID-associated cognitive impairment. Nat. Neurosci..

[100] Verveen A., Wynberg E., van Willigen H.D.G., Boyd A., de Jong M.D., de Bree G., Davidovich U., Lok A., Moll van Charante E.P., Knoop H. (2022). Severe Fatigue in the First Year Following SARS-CoV-2 Infection: A Prospective Cohort Study. Open Forum Infect. Dis..

[101] Azzolino D., Cesari M. (2022). Fatigue in the COVID-19 pandemic. Lancet Healthy Longev..

[102] Alghamdi H.Y., Alrashed A.M., Jawhari A.M., Abdel-Moneim A.S. (2022). Neuropsychiatric symptoms in post-COVID-19 long haulers. Acta Neuropsychiatr..

[103] Benedetti F., Palladini M., Paolini M., Melloni E., Vai B., De Lorenzo R., Furlan R., Rovere-Querini P., Falini A., Mazza M.G. (2021). Brain correlates of depression, post-traumatic distress, and inflammatory biomarkers in COVID-19 survivors: A multimodal magnetic resonance imaging study. Brain Behav. Immun.—Health.

[104] Moghimi N., Di Napoli M., Biller J., Siegler J.E., Shekhar R., McCullough L.D., Harkins M.S., Hong E., Alaouieh D.A., Mansueto G. (2021). The Neurological Manifestations of Post-Acute Sequelae of SARS-CoV-2 infection. Curr. Neurol. Neurosci. Rep..

[105] Zawilska J.B., Kuczyńska K. (2022). Psychiatric and neurological complications of long COVID. J. Psychiatr. Res..

[106] Felger J.C. (2019). Role of Inflammation in Depression and Treatment Implications. Handb. Exp. Pharmacol..

[107] de Mello A.J., Moretti M., Rodrigues A.L.S. (2022). SARS-CoV-2 consequences for mental health: Neuroinflammatory pathways linking COVID-19 to anxiety and depression. World J. Psychiatry.

[108] Chamberlain S.R., Cavanagh J., de Boer P., Mondelli V., Jones D.N.C., Drevets W.C., Cowen P.J., Harrison N.A., Pointon L., Pariante C.M. (2019). Treatment-resistant depression and peripheral C-reactive protein. Br. J. Psychiatry J. Ment. Sci..

[109] Dantzer R., O’Connor J.C., Lawson M.A., Kelley K.W. (2011). Inflammation-associated depression: From serotonin to kynurenine. Psychoneuroendocrinology.

[110] Liu J.J., Wei Y.B., Strawbridge R., Bao Y., Chang S., Shi L., Que J., Gadad B.S., Trivedi M.H., Kelsoe J.R. (2020). Peripheral cytokine levels and response to antidepressant treatment in depression: A systematic review and meta-analysis. Mol. Psychiatry.

[111] Chee Y.J., Fan B.E., Young B.E., Dalan R., Lye D.C. (2023). Clinical trials on the pharmacological treatment of long COVID: A systematic review. J. Med. Virol..

[112] Gorenshtein A., Liba T., Leibovitch L., Stern S., Stern Y. (2024). Intervention modalities for brain fog caused by long-COVID: Systematic review of the literature. Neurol. Sci..

[113] Asadi-Pooya A.A., Akbari A., Emami A., Lotfi M., Rostamihosseinkhani M., Nemati H., Barzegar Z., Kabiri M., Zeraatpisheh Z., Farjoud-Kouhanjani M. (2022). Long COVID syndrome-associated brain fog. J. Med. Virol..

[114] Krishnan K., Lin Y., Prewitt K.-R.M., Potter D.A. (2022). Multidisciplinary Approach to Brain Fog and Related Persisting Symptoms Post COVID-19. J. Health Serv. Psychol..

[115] Miller A., Song N., Sivan M., Chowdhury R., Burke M.R. (2025). Exploring the experiences of cognitive symptoms in Long COVID: A mixed-methods study in the UK. BMJ Open.

[116] Hartung T.J., Neumann C., Bahmer T., Chaplinskaya-Sobol I., Endres M., Geritz J., Haeusler K.G., Heuschmann P.U., Hildesheim H., Hinz A. (2022). Fatigue and cognitive impairment after COVID-19: A prospective multicentre study. EClinicalMedicine.

[117] Delgado-Alonso C., Valles-Salgado M., Delgado-Álvarez A., Yus M., Gómez-Ruiz N., Jorquera M., Polidura C., Gil M.J., Marcos A., Matías-Guiu J. (2022). Cognitive dysfunction associated with COVID-19: A comprehensive neuropsychological study. J. Psychiatr. Res..

[118] Hameed R., Bahadur A.R., Singh S.B., Sher J., Todua M., Moradi L., Bastakoti S., Arslan M., Ajmal H., Lee G.Y. (2023). Neurological and Psychiatric Manifestations of Long COVID-19 and Their [18F]FDG PET Findings: A Review. Diagnostics.

[119] Aretouli E., Malik M., Widmann C., Parker A.M., Oh E.S., Vannorsdall T.D. (2025). Cognitive and mental health outcomes in long covid. BMJ.

[120] Panagea E., Messinis L., Patrikelis P., Malefaki S., Petri M.C., Nasios G., Liontos A., Biros D., Kosmidis M.H., Milionis H. (2025). Persistent neuropsychological deficits in recovered COVID-19 patients: Correlations with disease biomarkers. Appl. Neuropsychol. Adult.

[121] Graham E.L., Clark J.R., Orban Z.S., Lim P.H., Szymanski A.L., Taylor C., DiBiase R.M., Jia D.T., Balabanov R., Ho S.U. (2021). Persistent neurologic symptoms and cognitive dysfunction in non-hospitalized Covid-19 “long haulers”. Ann. Clin. Transl. Neurol..

[122] Volk P., Rahmani Manesh M., Warren M.E., Besko K., Gonçalves de Andrade E., Wicki-Stordeur L.E., Swayne L.A. (2024). Long-term neurological dysfunction associated with COVID-19: Lessons from influenza and inflammatory diseases?. J. Neurochem..

[123] Toepfner N., Brinkmann F., Augustin S., Stojanov S., Behrends U. (2024). Long COVID in pediatrics-epidemiology, diagnosis, and management. Eur. J. Pediatr..

[124] Lopez-Leon S., Wegman-Ostrosky T., Ayuzo Del Valle N.C., Perelman C., Sepulveda R., Rebolledo P.A., Cuapio A., Villapol S. (2022). Long-COVID in children and adolescents: A systematic review and meta-analyses. Sci. Rep..

[125] Sousa I.P., de Sequeira P.C., Marcos A.C.S.B., Almeida N.A.A., Huff H.V., Solomon T., de Paula V.S. (2025). The importance of long-term studies in children following viral infection of the central nervous system. eClinicalMedicine.

[126] Reis P.A., Castro-Faria-Neto H.C. (2022). Systemic Response to Infection Induces Long-Term Cognitive Decline: Neuroinflammation and Oxidative Stress as Therapeutical Targets. Front. Neurosci..

[127] He D., Yuan M., Dang W., Bai L., Yang R., Wang J., Ma Y., Liu B., Liu S., Zhang S. (2023). Long term neuropsychiatric consequences in COVID-19 survivors: Cognitive impairment and inflammatory underpinnings fifteen months after discharge. Asian J. Psychiatry.

[128] Anjum A., Hussain A. (2025). Post-COVID Neurocognitive Disorder and Its Relation with Interleukin: A Hospital-based Cross-sectional Study. Indian J. Psychol. Med..

[129] Patrascu R., Dumitru C.S. (2025). Advances in Understanding Inflammation and Tissue Damage: Markers of Persistent Sequelae in COVID-19 Patients. J. Clin. Med..

[130] Franke C., Boesl F., Goereci Y., Gerhard A., Schweitzer F., Schroeder M., Foverskov-Rasmussen H., Heine J., Quitschau A., Kandil F.I. (2023). Association of cerebrospinal fluid brain-binding autoantibodies with cognitive impairment in post-COVID-19 syndrome. Brain Behav. Immun..

[131] Wongchitrat P., Chanmee T., Govitrapong P. (2024). Molecular Mechanisms Associated with Neurodegeneration of Neurotropic Viral Infection. Mol. Neurobiol..

[132] Fries G.R., Pfaffenseller B., Stertz L., Paz A.V.C., Dargél A.A., Kunz M., Kapczinski F. (2012). Staging and neuroprogression in bipolar disorder. Curr. Psychiatry Rep..

[133] Wilkowska A., Cubała W.J. (2022). The Downstaging Concept in Treatment-Resistant Depression: Spotlight on Ketamine. Int. J. Mol. Sci..

[134] Strong M.J. (2022). SARS-CoV-2, aging, and Post-COVID-19 neurodegeneration. J. Neurochem..

[135] Kamal M.A. (2022). Link of COVID-19 and Neurodegenerative Disorders. CNS Neurol. Disord.—Drug Targets.

[136] Zhang Y., Bi K., Zhou L., Wang J., Huang L., Sun Y., Peng G., Wu W. (2024). Advances in Blood Biomarkers for Alzheimer’s Disease: Ultra-Sensitive Detection Technologies and Impact on Clinical Diagnosis. Degener. Neurol. Neuromuscul. Dis..

[137] Yang L., Kim T.W., Han Y., Nair M.S., Harschnitz O., Zhu J., Wang P., Koo S.Y., Lacko L.A., Chandar V. (2024). SARS-CoV-2 infection causes dopaminergic neuron senescence. Cell Stem Cell.

[138] Khatoon F., Prasad K., Kumar V. (2022). COVID-19 associated nervous system manifestations. Sleep Med..

[139] Dolatshahi M., Sabahi M., Aarabi M.H. (2021). Pathophysiological Clues to How the Emergent SARS-CoV-2 Can Potentially Increase the Susceptibility to Neurodegeneration. Mol. Neurobiol..

[140] Piekut T., Hurła M., Banaszek N., Szejn P., Dorszewska J., Kozubski W., Prendecki M. (2022). Infectious agents and Alzheimer’s disease. J. Integr. Neurosci..

[141] Frost G.R., Jonas L.A., Li Y.-M. (2019). Friend, Foe or Both? Immune Activity in Alzheimer’s Disease. Front. Aging Neurosci..

[142] Ferini-Strambi L., Salsone M. (2021). COVID-19 and neurological disorders: Are neurodegenerative or neuroimmunological diseases more vulnerable?. J. Neurol..

[143] Rodriguez-Perez A.I., Garrido-Gil P., Pedrosa M.A., Garcia-Garrote M., Valenzuela R., Navarro G., Franco R., Labandeira-Garcia J.L. (2020). Angiotensin type 2 receptors: Role in aging and neuroinflammation in the substantia nigra. Brain. Behav. Immun..

[144] Gomez-Pinedo U., Matias-Guiu J., Sanclemente-Alaman I., Moreno-Jimenez L., Montero-Escribano P., Matias-Guiu J.A. (2020). Is the brain a reservoir organ for SARS-CoV2?. J. Med. Virol..

[145] Naughton S.X., Raval U., Pasinetti G.M. (2020). Potential Novel Role of COVID-19 in Alzheimer’s Disease and Preventative Mitigation Strategies. J. Alzheimer’s Dis..

[146] Fazzini E., Fleming J., Fahn S. (1992). Cerebrospinal fluid antibodies to coronavirus in patients with Parkinson’s disease. Mov. Disord..

[147] Antonini A., Leta V., Teo J., Chaudhuri K.R. (2020). Outcome of Parkinson’s Disease Patients Affected by COVID-19. Mov. Disord..

[148] Marreiros R., Müller-Schiffmann A., Trossbach S.V., Prikulis I., Hänsch S., Weidtkamp-Peters S., Moreira A.R., Sahu S., Soloviev I., Selvarajah S. (2020). Disruption of cellular proteostasis by H1N1 influenza A virus causes α-synuclein aggregation. Proc. Natl. Acad. Sci. USA.

[149] Ait Wahmane S., Achbani A., Ouhaz Z., Elatiqi M., Belmouden A., Nejmeddine M. (2020). The Possible Protective Role of α-Synuclein Against Severe Acute Respiratory Syndrome Coronavirus 2 Infections in Patients with Parkinson’s Disease. Mov. Disord..

[150] Iravanpour F., Farrokhi M.R., Jafarinia M., Oliaee R.T. (2023). The effect of SARS-CoV-2 on the development of Parkinson’s disease: The role of α-synuclein. Hum. Cell.

[151] Etter M.M., Martins T.A., Kulsvehagen L., Pössnecker E., Duchemin W., Hogan S., Sanabria-Diaz G., Müller J., Chiappini A., Rychen J. (2022). Severe Neuro-COVID is associated with peripheral immune signatures, autoimmunity and neurodegeneration: A prospective cross-sectional study. Nat. Commun..

[152] Peluso M.J., Deeks S.G. (2024). Mechanisms of long COVID and the path toward therapeutics. Cell.

[153] Zhang H., Zang C., Xu Z., Zhang Y., Xu J., Bian J., Morozyuk D., Khullar D., Zhang Y., Nordvig A.S. (2023). Data-driven identification of post-acute SARS-CoV-2 infection subphenotypes. Nat. Med..

[154] Gottlieb M., Spatz E.S., Yu H., Wisk L.E., Elmore J.G., Gentile N.L., Hill M., Huebinger R.M., Idris A.H., Kean E.R. (2023). Long COVID Clinical Phenotypes up to 6 Months After Infection Identified by Latent Class Analysis of Self-Reported Symptoms. Open Forum Infect. Dis..

[155] Parhizgar P., Yazdankhah N., Rzepka A.M., Chung K.Y.C., Ali I., Lai Fat Fur R., Russell V., Cheung A.M. (2023). Beyond Acute COVID-19: A Review of Long-term Cardiovascular Outcomes. Can. J. Cardiol..

[156] Panda M., Kalita E., Singh S., Kumar K., Rao A., Prajapati V.K. (2022). MiRNA-SARS-CoV-2 dialogue and prospective anti-COVID-19 therapies. Life Sci..

